# Comparing cisplatin-Chemoradiotherapy to Cetuximab-radiotherapy in HPV^+^ “low-risk” locally advanced oropharyngeal squamous cell carcinoma: lessons from De-escalate study

**DOI:** 10.1186/s41199-019-0040-5

**Published:** 2019-03-12

**Authors:** Panagiota Economopoulou, Amanda Psyrri

**Affiliations:** National Kapodistrian University of Athens, Attikon Hospital, Rimini 1, 12461 Athens, Greece

**Keywords:** Hpv, Oropharyngeal cancer, Cetuximab, Radiotherapy, de-escalate

## Abstract

**Background:**

Human papillomavirus-associated oropharyngeal cancer (HPV-OSCC) is rapidly increasing in incidence and has unique epidemiologic, molecular, and biologic characteristics. Standard combined modality therapies for head and neck cancer confer a significant risk of morbidity. However, patients with HPV-OSCC are diagnosed at a younger age and have a superior prognosis; this spurs the development of treatment deintensification trials that attempt to decrease treatment-related morbidity without compromising efficacy.

**Main body:**

The De-Escalate-HPV is a randomized phase 3 study that compares the standard treatment, radiation and cisplatin, with radiation and epidermal growth factor receptor (EGFR) inhibitor cetuximab in patients with low-risk HPV-OSCC.

**Conclusion:**

In this commentary, we aim to discuss the results of the De-Escalate-HPV study.

## Background

Human papillomavirus (HPV)-associated oropharyngeal squamous cell carcinoma (HPV-OSCC) represents a distinct disease entity that is characterized by markedly improved survival [1]. The current standard of care for HPV-OSCC is derived from older trials of head and neck cancer patients with predominately HPV-negative disease, potentially representing overtreatment of favorable-risk, HPV-positive patients. However, given the different etiology, natural history, molecular landscape and treatment responsiveness, it is now accepted that HPV-positive and HPV-negative OSCCs are distinct diseases [2]. Consequently, current trials in HPV-OSCC seek to examine treatment de-escalation strategies aiming to minimize morbidity and avoid exposure of those patients to overtreatment.

## Main text

A main de-escalation strategy carried out for patients with HPV-OSCC is investigation of cetuximab as an alternative to cisplatin in combination with intensity modulated radiation therapy (IMRT), attempting to reduce cisplatin late effects such as neuropathy, nephropathy and ototoxicity [3]. In this context, the main purpose of the phase III randomized De-Escalate HPV trial, which is reported in the current issue of Lancet, was to compare the severe acute and late toxicity caused by cetuximab and radiotherapy (RT) to that caused by cisplatin and RT in patients with low-risk HPV-OSCC [4]. Secondary endpoints included overall survival (OS) and recurrence rates between treatment arms. In comparison to cisplatin, cetuximab was not found to be superior in terms of overall toxicity; however, it was shown to have a distinct spectrum of toxicities and less serious adverse events. Interestingly, cetuximab and radiation resulted in worse OS, locoregional and distant control.

The overall principle guiding treatment is “primum non nocere” (first, to do no harm); thus, treatment de-intensification is only conceivable in “low risk” patients, compromising patient’s safety being unacceptable. Indeed, the De-Escalate HPV Trial focuses on low-risk “favorable” HPV-OSCC subgroup, based on American Joint Committee on Cancer/International Union for Cancer Control [AJCC/UICC] tumor, node, and metastasis [TNM] 7th Edition manual (T3 N0–T4 N0, and T1 N1–T4 N3) and classification by Ang et al. in their retrospective analysis of the Radiation Therapy Oncology Group (RTOG) 0129 study cohort, in which 63.8% of patients with stage III–IV OSCC were found to have HPV-associated cancers [5]. In this study, the risk of death increased significantly with each additional pack-year of smoking. Patients were thus grouped by risk: those with HPV-associated disease and ≤ 10 pack-year smoking history as well as those with HPV-associated disease, > 10 pack-year smoking history, and N0–N2a disease were deemed low-risk, with a 3-year OS of 93% [5]. Therefore, patient eligibility of the De-Escalate HPV trial was based on a non-HPV-OSCC specific staging system and a “low risk” subgroup definition derived from a recursive-partitioning analysis of a small OSCC cohort; this raises questions about appropriate selection of patients included in the study. Nevertheless, there is still no consensus on which clinical and biological parameters to consider for selection of patients with HPV-OSCC and good prognosis. The American Joint Committee on Cancer eighth edition staging system devoted to HPV-driven OSCC might potentially help to properly select patients for treatment de-escalation. This new classification better differentiates prognosis of patients according to their TNM stage. Of note, smoking history is not taken into account. On the other hand, variations in HPV biology might give insight into patient risk stratification. In a recent report by Gleber-Netto et al., researchers analyzed data from 80 oropharyngeal cancers in The Cancer Genome Atlas and found a panel of 582 HPV-correlated genes that distinguished three subgroups: a high-HPV group, a low-HPV group, and an HPV-negative group. Each group had statistically significant survival differences. Additional analysis led to a panel of 38 genes that are able to distinguish between the two HPV-positive subgroups. Interestingly, two viral genes (E1 and E1^E4) were significantly different between these subgroups and E1^E4 cell-lines were more radiosensitive. Based on gene panel expression, researchers developed a prognostic and potentially predictive biomarker signature associated with HPV function; the gene panel appeared to be prognostic of survival and performed better than available clinical factors [6]. Indeed, incorporation of molecular markers into patient selection could lead to safer implementation of de-intensified treatment protocols and facilitate testing of new treatment approaches for patients with unresponsive tumors.

The primary endpoint of the De-Escalate HPV trial was the overall acute and late severe toxicity between treatment arms. It was demonstrated that cisplatin and cetuximab have the same rates of severe and overall acute and late toxicity; however, cisplatin was found to cause more serious adverse events. More specifically, 162 adverse events occurred in patients receiving cisplatin and 95 events occurred in patients receiving cetuximab (*p* < 0·0001). The most common serious adverse events for cisplatin were vomiting (in 30% of patients) and nausea (in 28% of patients), and those for cetuximab were vomiting (13%) and oral mucositis (13%). In addition, serious adverse events in the cisplatin group were more likely to be assessed as related or possibly related to treatment (68%) than in the cetuximab group (19%). Interestingly, in the Bonner trial, severe toxicity of cetuximab plus RT was similar to RT alone, with the exception of skin rash [7]. On the other hand, in the phase II TREMPLIN trial that compared cisplatin-based chemoradiotherapy versus radiotherapy in combination with cetuximab for larynx preservation, any grade acute toxicity was significantly higher in the cisplatin arm, albeit late toxicity did not differ between treatment arms [8]. The safety results of the De-Escalate HPV trial must be interpreted with caution; in fact, only 38.3% of patients received the full chemotherapy protocol in the cisplatin arm compared to 79% in the cetuximab arm, the main reasons for discontinuation/reduction in cisplatin dose being myelosuppression or gastrointestinal toxicity; thus, comparison may not be accurate due to compliance differences. In addition, although quality of life was assessed by a questionnaire specific for head and neck cancer, head and neck module scales were not analyzed. Moreover, the primary endpoint was not met in this trial; failure to reject the null hypothesis (no difference in toxicity rates between cisplatin and cetuximab) should not be considered as a proof of equivalence between the two treatment arms. Nevertheless, taking into account that cisplatin-based chemoradiation remains the standard of care, the higher rate of serious adverse events and poor compliance in the cisplatin arm underlines the significance of supportive measures to reduce treatment-related symptom burden.

Although OS was a secondary endpoint in this trial, a marked difference in OS was noted in favor of cisplatin (2 year OS 97.5% vs. 89.4%, *p* = 0.001, HR = 4.99, CI 1.70–14.67). In addition, the 2-year recurrence rate was higher in patients treated with cetuximab (6·0% vs 16·1%, 3·4 [1·6–7·2]; log-rank *p* = 0·0007). Interestingly, survival remained significantly different after adjusting for prognostic factors (T4, N3) although the high number of statistical comparisons performed increases dramatically the probability of type I error. Given that efficacy outcomes are secondary, power of statistical significance is missing. In addition, during follow up, there were only 26 deaths with 19 cancer-related deaths. Thus, follow up is likely inadequate to warrant a stable OS estimate, rendering survival data immature. Nevertheless, the randomized phase III study 1016 conducted by RTOG that was recently published provided definitive evidence of a survival difference between cisplatin-based and cetuximab-based radiotherapy [9]. RTOG 1016 was a non-inferiority trial which compared cisplatin chemoradiotherapy to cetuximab and radiation in patients with locally advanced HPV-OSCC. Primary endpoint was OS. After median follow-up duration of 4.5 years, radiotherapy plus cetuximab did not meet the non-inferiority criteria for OS (HR = 1·45, one-sided 95% upper CI 1·94; *p* = 0·5056 for non-inferiority; one-sided log-rank *p* = 0·0163); indeed estimated 5-year OS was higher in the cisplatin arm [84.6% (96% CI 80·6–88·6) vs.77·9% (95% CI 73·4–82·5) in the cetuximab arm] [9]. Although direct comparisons between trials cannot be made, RTOG 1016 included more patients (849 vs.334 in the De-Escalate trial), did not focus on “low risk” HPV-OSCC, used a different cisplatin chemotherapy regimen (omission of the 3rd cycle) and a different radiation schedule, had OS as the primary endpoint and results were reported after 5 years of follow-up versus 26 months in the De-Escalate HPV trial. Regarding toxicity, in the RTOG 1016 trial, although the overall proportion of one or more grade 3–4 acute adverse events was similar in the cetuximab and cisplatin groups (77.4% vs. 81.7%; *p* = 0.16), several adverse events such as myelosuppression, anemia, nausea, vomiting, anorexia, dehydration, hyponatremia, kidney injury, and hearing impairment were significantly more frequent in the cisplatin group, whereas only acneiform rash was significantly more frequent in the cetuximab group.

Of note, the type of radiotherapy used in the De-Escalate HPV trial was once daily fractionation. In the Bonner trial, investigators were allowed to select between once daily-fractionation, twice-daily fractionation and concomitant boost radiotherapy. In the cetuximab arm, the effect of treatment on the duration of survival was more prominent in the combined radiotherapy treatment (concomitant boost). Based on this observation, one could argue that the reduced efficacy observed in the cetuximab arm in the De-Escalate HPV trial might be in part related to the type of radiotherapy used. However, in the Bonner trial, the study was not powered to detect differences among radiotherapy subgroups.

Prudential evaluation of the De-Escalate trial raises several questions. Should this be the end of de-intensification strategies in locally advanced HPV- OSCC? Should we re-think the use of targeted agents in the absence of predictive biomarkers? On a mechanistic level, the existing data are conflicting. Studies have shown an inverse correlation between HPV status and Epidermal EGFR alterations [10]. Moreover, integrative analyses of gene expression and gene copy numbers have shown that HPV-driven OSCC is characterized by a lack of Epidermal Growth Factor (EGFR) protein expression or gene amplification [11]. Furthermore, predictors of response to cetuximab in head and neck cancer have not been identified. The most important key to answering these extremely important questions is further investigation for the development of predictive biomarkers that will guide treatment selection.

## Conclusions

In conclusion, cisplatin chemoradiotherapy remains the standard of care for stages III-IVA HPV-OSCC. Well-designed randomized clinical trials testing novel non-toxic therapies against cisplatin in biomarker-enriched populations should be conducted in order to guide patient selection and improve de-intensification strategies.

## Abbreviations

AJCC: American Joint Committee on Cancer
EGFR: Epidermal Growth Factor Receptor
HPV: Human Papillomavirus
HPV-OSCC: HPV-associated oropharyngeal squamous cell carcinoma
HR: Hazard Ratio
IMRT: Intensity-Modulated Radiation Therapy
OS: Overall survival
OSCC: Oropharyngeal squamous cell carcinoma
RTOG: Radiation Oncology Group
TNM: Tumor, node and metastasis
UICC: International Union for Cancer Control
